# Aerobic but not Resistance Exercise Can Induce Inflammatory Pathways via Toll-Like 2 and 4: a Systematic Review

**DOI:** 10.1186/s40798-017-0111-2

**Published:** 2017-11-28

**Authors:** Paula Andréa Malveira Cavalcante, Marcos Fernandes Gregnani, Jessica Salles Henrique, Fábio Henrique Ornellas, Ronaldo Carvalho Araújo

**Affiliations:** 1 Medicine (Nephrology) Program, Federal University of São Paulo (UNIFESP), São Paulo, SP Brazil; 2 Molecular Biology Program, Federal University of São Paulo (UNIFESP), São Paulo, SP Brazil; 3 Laboratory of Exercise Genetics and Metabolism, Federal University of São Paulo (UNIFESP), São Paulo, SP Brazil; 4 Department of Biophysics, Federal University of São Paulo (UNIFESP), São Paulo, SP Brazil; 5 Neurology/Neuroscience Program, Federal University of São Paulo (UNIFESP), São Paulo, SP Brazil; 6 Exercise Neurophysiology Laboratory, Federal University of São Paulo (UNIFESP), São Paulo, SP Brazil; 7 Rua Pedro de Toledo, 669/9and., 04039-032, São Paulo, SP Brazil

**Keywords:** TLR2, TLR4, Toll-like, Exercise, Training, Aerobic, Resistance, Inflammation

## Abstract

**Background:**

Only a few studies have addressed the relationship between toll-like receptors 2 and 4 (TLR2 and TLR4) and the production of local and systemic cytokines in response to physical exercise, and they have produced conflicting results. We aimed to determine whether acute and chronic exercise outcomes are associated with changes in TLR2 and TLR4 expression and signaling and if so, the mechanisms that connect them.

**Methods:**

PubMed database were consulted. This systematic review selected 39 articles, 26 involving humans and 13 based on rodents.

**Results:**

In acute resistance exercise studies, 75% reported a decrease in TLR4 or TLR2 expression and 25% did not find differences. For chronic resistance exercise studies, 67% reported a reduction of expression and 33% did not find differences. Studies of both types reported reductions in pro-inflammatory cytokines. In acute aerobic exercise studies, 40% revealed a decline in the expression of the receptors, 7% reported no significant difference, 40% showed an increase, and 13% did not evaluate their expression. Fifty-eight percent of studies of chronic aerobic exercise revealed a reduction in expression, 17% did not find a difference, and 25% reported increases; they also suggested that the expression of the receptors might be correlated with that of inflammatory cytokines. In studies on combined exercise, 50% reported a decline in receptors expression and 50% did not find a difference.

**Conclusions:**

The majority of the articles (54%) link different types of exercise to a decline in TLR4 and TLR2 expression. However, aerobic exercise may induce inflammations through its influence on these receptor pathways. Higher levels of inflammation were seen in acute sessions (40%) than regular sessions (25%).

## Key Points

- It is known that regular exercise acts as an anti-inflammatory agent by down-regulating TLR4 in immune cells. Paradoxically, acute, extended, or intense exercise can be harmful to the immune system.
- The molecular mechanisms by which various types of physical exercise modulate the TLR2 and TLR4 pathways are still not fully understood.
- Physical exercise reduced the expression of TLR2 and TLR4. However, aerobic exercise is potentially inflammatory when compared with resistance exercise.

## Background

The connections between lifestyle factors and health have been the subject of intense research, partly motivated by alarming changes in the health landscape of industrialized societies. One clear trend is that moderate exercise benefits health in many ways, while extremes of too little or excessive exercise have been linked to chronic diseases. Many of these have an immune component—individuals with very sedentary lifestyles often fall prey to low-grade chronic inflammations [1–4]. Over the long term, this condition can lead to type 2 diabetes, cardiovascular diseases, particular types of cancer, chronic respiratory diseases, and other serious health problems. Physicians have called this constellation a worldwide epidemic [5]. The immune system can also be disrupted by excessive exercise. While progress has been made, there remain many gaps in our understanding of the mechanisms that connect the types and amounts of a person’s activity to immune responses and disease.

The prevalence of inflammations suggests a logical point of departure for such studies. Inflammation involves complex interactions at the molecular and cellular levels that can arise in any vascular tissue as a result of traumatic, infectious, post-ischemic, toxic, or autoimmune injuries [6]. Toll-like receptors play a role in many of these conditions; they are known to make significant contributions to obesity [7, 8], type 2 diabetes [9], non-alcoholic steatosis [10], cardiovascular disease [11, 12], cerebral ischemia [13, 14], Alzheimer’s disease [15], rheumatoid arthritis [16], and other diseases. This review examined recent work that suggests they also help modulate the effects of different levels of physical activity on states of health and disease.

TLRs are type I transmembrane proteins involved in both innate and adaptive immune system responses [17, 18]. These receptors mediate the recognition of pathogen-associated molecular patterns (PAMPs) or damage-associated molecular patterns (DAMPs)—specific molecules released by damaged or necrotic cells [18, 19]. The immune activities of TLRs are generally modulated through signaling via the NF-kB pathway. Responses begin with the stimulation of the receptor by an external signal. This alters the cytoplasmic regions of TLRs, which contain Toll/interleukin-1 (IL-1) receptor (TIR) domains. Stimulation causes these domains to recruit adaptor proteins in a process that ultimately activates the nuclear transcription factor NF-kB [17]. This releases NF-kB for transport to the cell nucleus, where it triggers the transcription of cytokines including IL-1β, IL-6, and IL-8 interleukins; TNF-α [20–22]; and other elements [23] that play key roles in the immune system responses. Alongside cytokines, NF-kB induces the expression of growth factors and other molecules involved in stress response, cell proliferation, and cell cycle progression [24–26].

TLRs are expressed in the immune cells including macrophages, dendritic cells (DCs), B cells, and specific types of T cells. They are also present in non-immune cells such as fibroblasts and epithelial cells [27] and in the tissues of the ovary, prostate, placenta, testicles, lungs, liver, and skeletal muscle [28].

The toll-like receptors TLR2 and TLR4 have received particular attention due to their ability to identify molecular patterns exhibited by several invasive pathogens [18]. They also seem to play an important role in the anti-inflammatory effects observed in physically active individuals [29]. Regular exercise has been determined to have anti-inflammatory effects [2, 29–34] by downregulating TLR4 in the immune cells. A bit paradoxically, at the other end of the activity spectrum, acute, extended, or intense exercise can have a negative impact on the immune system [35–42]. But the molecular mechanisms by which exercise modulates the TLR2 and TLR4 pathways are still not fully understood.

One plausible link comes from the demonstration that TLR2 and TLR4 are activated by the extracellular non-esterified fatty acids (NEFAs). Concentrations of extracellular NEFAs undergo transient increases during aerobic exercise (AE). If levels are chronically elevated, however, TLRs may induce the production of pro-inflammatory cytokines in macrophages, adipocytes, liver, and skeletal muscle cells. This suggests that the receptors may participate in the development of insulin resistance [43]. Yet, they also have protective effects against insulin resistance, which may be explained by the down-regulation of TLR expression that occurs during physical exercise [43].

Here, this review investigated the existing literature on the inflammatory and anti-inflammatory effects of different types of physical exercise with a focus on systematically collecting connections to TLR2 and TLR4 modulation and signaling. To accomplish this, the results were divided into single sessions of acute exercise and chronic exercise, based on periodicity. Additionally, this review identified key biomarkers and analyzed the combined TLR2 and TLR4 responses to markers involved in the process of inflammation process, including anti- and pro-inflammatory cytokines, adaptor proteins, and the transcription factor NF-kB.

### Inflammatory Effects of Physical Exercise

Analyzing the modulation of inflammation patterns permits insights into specific underlying physiological mechanisms. As a controllable model of stress, physical exercise is a good tool to analyze inflammatory responses [44].

Physical exercise permits the control of variables related to activity such as volume, intensity, frequency, and duration. These factors have led to its adoption as a good strategy to study alterations that occur due to inflammations caused by stress and their implications for health [45–47]. Local and systemic cytokine production in response to physical exercise resembles the cytokine response to infections, trauma, and sepsis [44, 45, 48]. There is evidence that very strenuous physical exercise can cause substantial tissue damage and initiate an inflammatory reaction and excessive immunosuppression, in a way that highly resembles features observed in clinical sepsis [49]. However, trauma, infection, and septic complications can produce an uncontrollable inflammatory response with long-term detrimental or fatal consequences. In physical exercise, although the inflammatory cascade has obvious similarities, the response appears to be limited [44].

Usually, the process of inflammation has an overall positive effect on the organism. Short-term, acute inflammation allows the body to survive progressive tissue destruction by promoting healing [50, 51]. On the other hand, if destruction and repair are not properly coordinated, inflammation may lead to persistent tissue damage. The mechanisms by which acute inflammation starts and develops are well understood, but little is known about the causes of chronic inflammation and its association with molecular and cellular pathways [51].

A comparison can also be made between chronic inflammation and strenuous physical exercise in which pro-inflammatory pathways seem to be activated [38, 41, 52]. In response to heavy exercise, inflammation stimulates tissue monocyte production, and platelet hyperactivity promotes fibrinogen biosynthesis and induces the formation of the microparticle and the accumulation of erythrocytes to trigger a prothrombotic state. In fact, vigorous aerobic exercise may be atherogenic and atherothrombotic due to the overproduction of mitochondrial-free radicals in the skeletal and myocardial muscle. On the other hand, both moderate AE and low-load resistance exercise (RE) may reduce inflammation and improve fibrinolysis. [52].

An elegant study [53] found associations between all causes of mortality and doses of jogging. Light and moderate joggers had a lower mortality than sedentary non-joggers, while there was no significant statistical difference between mortality in strenuous joggers and the sedentary group. In this analysis, high running loads in sports such as marathons, ultramarathons, triathlons, and long high-intensity bike rides can cause negative effects such as acute inflammations; in the long term, these activities may lead to chronic inflammation, irregular fibrosis formation, alterations in the size of the cardiac chambers, and atrial fibrillation [54]. Moreover, long-distance runners may have increased levels of atherosclerosis and coronary disease due to constant training throughout the year [54]. In atherosclerosis, the endothelial permeability is increased by the oxidative damage that promotes the entry of lipoproteins in the subendothelial space, resulting in inflammation [55]. When the lipoproteins are oxidative, they interact with TLR4 in particular and promote cardiovascular disease [56].

According to the American College of Sports Medicine (ACSM) and the American Heart Association [57], the minimum recommendation for physical exercise for adults and seniors aiming to avoid chronic disease is 30 min of moderate aerobic activity per day, five times a week; 20 min per day of intense activity, three times a week; or a combination of moderate and vigorous activity. These guidelines also suggest that high loads of AE may be necessary for some groups to prevent a transition to an estimation that they are overweight or a diagnosis of obesity. However, they also recommend limiting vigorous physical training to 60 min a day, for a weekly total of no more than 5 h, including 1 to 2 days without high-intensity exercise per week [58, 59]. Strenuous AE has been shown to induce an excess of reactive oxygen species (ROS) [60]; can modulate TLR4 signal transduction at many levels [61]; stimulate pro-inflammatory transcription factors such as NF-kB, AP-1, and Nrf2 [62, 63]; and promote inflammation [64].

NADPH oxidase 4 (NOX4), involved in redox signaling in vascular cells, has direct interactions with TLR4 in both for the generation of endogenous and exogenous ROS-mediated by LPS and the activation of NF-kB [65]. In addition, high levels of ROS in the muscles can provoke a hyperactivation of the innate immune system in cells such as macrophages and neutrophils [66], and it leads to the production of several peroxides and aldehydes that are potentially toxic to the cells [67], also affecting T cell polarization and contributing to pro-inflammatory cytokine secretion [68]. It is already known that ROS production and neutrophil counts change in athletes involved in activities such as running, jumping, throwing, combined events (triathlon, heptathlon, and decathlon), swimming, cycling, and soccer, but only high-intensity exercise induces oxidative damage in lymphocytes [69]. In contrast, moderate-intensity AE stimulates the combat of excessive ROS by maintaining redox balance in the muscle [70]. A study [71] of soccer players showed a significant correlation between leukocyte ROS production and creatine kinase (CK) values, considered a qualitative marker for microtrauma skeletal muscle.

In fact, the physiological effects of strenuous AE, for example, participation in triathlons, include a large increase in CK, C-reactive protein (CRP), cortisol, and aldosterone and a decrease in testosterone levels [72]. Moreover, after strenuous exercise, increased levels of LPS may trigger an increase in the production of pro-inflammatory cytokines [73–76]. Long periods of AE [72] or short acute sessions of strenuous physical exercise [41] can disturb homeostasis and enhance inflammation. Consistent with this, Rodrigues-Miguelez et al. [39] found an increase in TLR4 and pro-inflammatory cytokines such as TNF-α and IL-1β in acute AE sessions; however, the effects were reversed with regular training in reasonable doses.

TNF-α represents a group of peptides that are released into the bloodstream in response to the endotoxin stimulation during infectious processes. TNF-α has a catabolic effect [77] and plays a role in the loss of muscle mass that usually appears in chronic diseases such as rheumatoid arthritis and cancer [78]. TNF-α genesis in low-grade systemic inflammation is thought to occur mainly in the adipose tissue [79–81]. Furthermore, systemic inflammation and high concentrations of pro-inflammatory cytokines act on the hypothalamic-pituitary-adrenal axis and can increase serum concentrations of cortisol [82, 83]. Physical exercise and nutrition modulate the cortisol response. Variables such as intensity, lactate accumulation, total volume, and resting period determine the level of cortisol released to stimulate glycogenolysis and gluconeogenesis [84, 85]. Moderate- to high-intensity exercise can cause increases in circulating levels of cortisol. On the other hand, low-intensity exercise (40% VO2max) reduces circulating levels of cortisol [84]. In the study by Lira et al. [76], TLR-4 and NF-kBp65 were increased in animals from both groups (overtraining and resting after overtraining). Additionally, a decrease in the performance and an increase in the production of corticosterone and endotoxin were observed in overtraining groups compared to both control and trained groups, indicating that chronically high levels of plasma cortisol can increase inflammation in the epididymal adipose tissue.

**Fig. 1:**
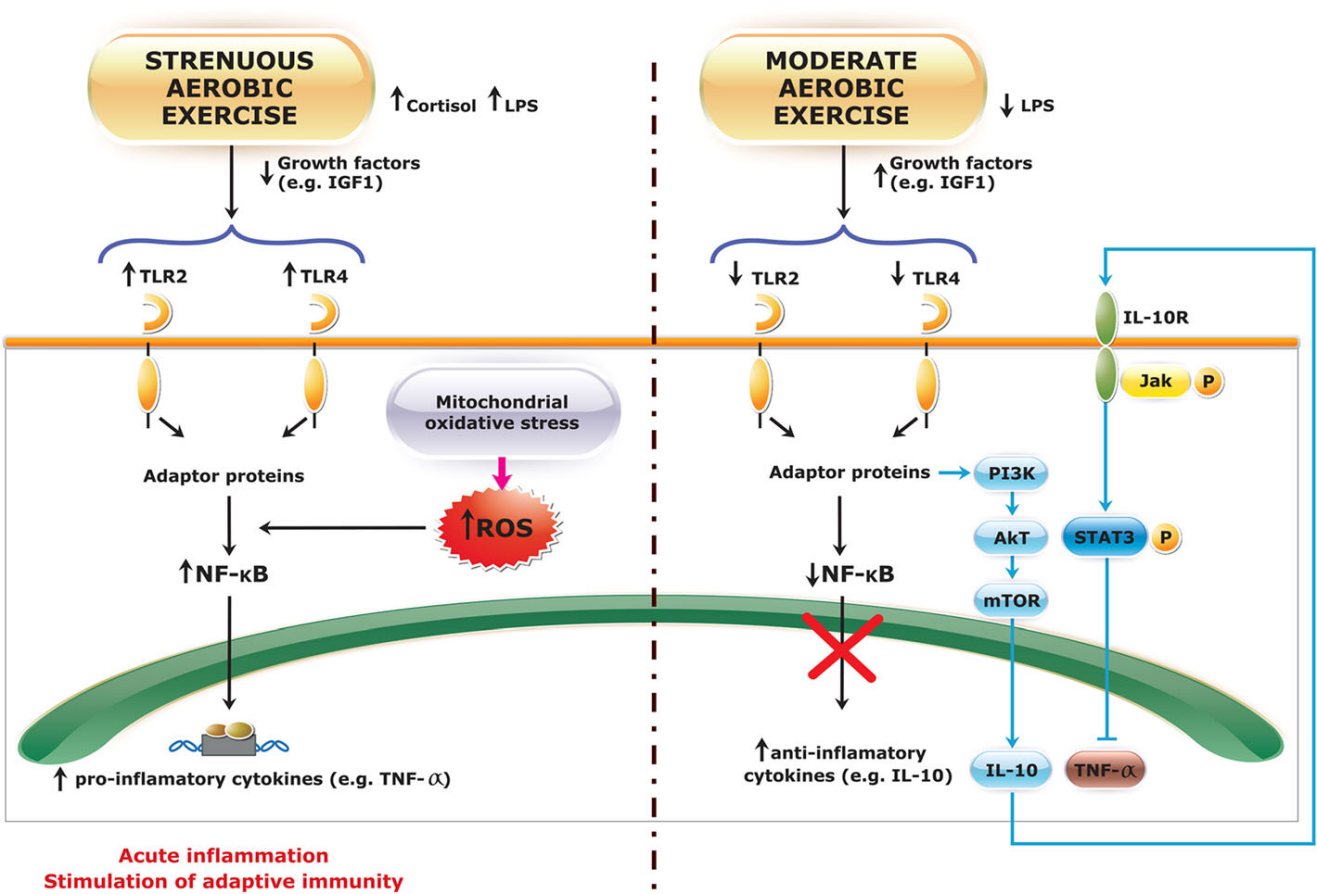
Signaling involving TLR2 and TLR4 in strenuous and moderate aerobic exercise. Excess physical exercise increases LPS levels and contributes to TLR2, TLR4, and NF-kB upregulation. As a consequence, there is an increase in circulating pro-inflammatory cytokines. Stimuli of exercise stress transmit nerve impulses to the brain, raising the levels of counter-regulatory hormones such as cortisol. Accordingly, high mitochondrial oxidative stress induced by strenuous aerobic exercise causes excessive intracellular ROS formation that also upregulates NF-kB expression, intensifying the acute inflammation state. Under these excessive stress conditions, adaptive immunity can be triggered by the increase in costimulatory molecules in antigen-presenting cells, thus activating T cells. In contrast, the regular physical exercise of moderate intensity reduces LPS, TLR2, TLR4, and NF-kB expression. Under these conditions, NF-kB does not translocate to the cell nucleus. Instead, the anti-inflammatory pathway PI3K/AKT/mTOR is activated, promoting the production of anti-inflammatory cytokines such as IL-10 that inactivate TNF-α. Physical exercise at a moderate intensity also has a compensatory effect against the exacerbated production of reactive oxygen and nitrogen species responsible for the oxidative damage. Elevated production of IGF-1 is observed after exercise. IGF-1 provides an anti-inflammatory effect on the skeletal muscle cells, reducing the expression of the pro-inflammatory cytokines through a decrease of TLR4 expression

Thereby, an excess of physical (blood cortisol levels) and oxidative stress (intracellular ROS accumulation) can generate temporary immune dysfunction [86]. In contrast, physical exercise at moderate intensities regulates the immune system and reduces oxidative stress [87]. Figure 1 presents a simplified comparison of some mechanisms that can be activated by strenuous physical exercise and by regular exercise performed at moderate intensity.

### Anti-inflammatory Effects of Physical Exercise

It is well known that regular physical exercise has anti-inflammatory effects [8, 29–31, 88–93]. Therefore, regular physical exercise, as well as a physically active lifestyle, may be useful as a treatment for a range of chronic diseases and conditions characterized by low-grade systemic inflammation [3, 94].

However, the link between physical exercise and TLRs is still a matter of debate. Although the pro-inflammatory effects of TLR2 and TLR4 signaling have been well studied, anti-inflammatory responses due to the activation of these receptors are still not fully understood [95]. For this reason, this article will briefly address a number of molecules that act directly during the processes of adaptation to physical exercise—including hormones, myokines, and chemical molecules such as ROS.

The skeletal muscle can function as an endocrine organ due to its production of growth hormones and cytokines known as myokines, which are induced by an exercise stimulus [96, 97]. One of the best-known exercise-induced adaptations [98, 99] is an increase in circulating levels of insulin-like growth factor 1 (IGF-1). Elevated levels of circulating IGF-1 have been observed after exercise, probably in response to hepatic secretion stimulated by growth hormone (GH) [85].

The first evidence that IGF-1 is a potent modulator of TLR4 (protein expression) in the skeletal muscles was provided by Lee [31]. The author demonstrated that IGF-1 stimulation had anti-inflammatory effects on the skeletal muscle and suppressed TLR4 signaling. Treatment with IGF-1 attenuated the amounts of endogenous IL-6 and TNF-α, indicating that IGF-1 had an anti-inflammatory effect on the skeletal muscle cells by reducing the expression of pro-inflammatory cytokines under baseline conditions through a down-regulation of the expression of TLR4. This led to a hypothesis that cells with low levels of TLR4 are less responsive to ligands that stimulate endogenous inflammation, such as the heat shock protein, and thus contribute to a lower basal response of pro-inflammatory cytokines [31]. In addition to the anti-inflammatory effects of IGF-1, regular AE promotes the remodeling of mitochondrial networks with significant improvements in both the quality and quantity of the mitochondria [100]. This results in positive changes in the respiratory capacity and oxygen extraction of trained subjects [100, 101].

Likewise, there is an increase in angiogenesis, the formation of new capillaries from pre-existing ones. High levels of VEGF—resulting from endurance training—offer favorable conditions for an increase in the density of the muscle capillaries [100]. Furthermore, a moderate level of AE reduces pro-atherogenic cytokines such as TNF-α and IFN-γ and simultaneously increases atheroprotective cytokines such as IL-4, IL-10, and TGF-β [102].

The anti-inflammatory effects of regular exercise might be mediated by a reduction of visceral fat mass followed by a decline in the release of adipocytokines, as well by the anti-inflammatory environment induced by exercise [103]. This environment consists of three variables: cortisol and adrenaline release from suprarenal glands, an increase in the production and release of IL-6 and other myokines from skeletal muscle, and a decrease in amounts of TLR (cell surface protein and mRNA expression) - in monocytes and macrophages, and as a consequence, the inhibition of the release of pro-inflammatory cytokines [103].

In fact, there is evidence that exercise is responsible for reducing the expression of these receptors at both mRNA expression and protein levels [2, 29, 30, 32, 93]. In diet-induced obesity rats (DIO), both acute aerobic exercise (AAE) and chronic aerobic exercise (CAE) led to a significant suppression of the TLR4 signaling pathway in liver, muscle, and adipose tissue, reduced LPS in serum, and improved insulin signaling [9]. However, the anti-inflammatory responses induced by TLR4 activation have not been characterized as clearly. In contrast to TLR4 pro-inflammatory signaling at the cell surface, TLR4 signaling from endosomal compartments induces the secretion of the anti-inflammatory cytokine IL-10 [95].

During physical exercise, a transient increase in IL-6 in circulation appears to be responsible for a further increase in the levels of circulating anti-inflammatory cytokines such as IL-10 and IL-1ra [104–106]; this also stimulates the release of cortisol from the adrenal glands [106]. Increases in IL-6 levels during exercise are transient and return to resting levels usually within 1 h after exercise [107]. This phenomenon may occur because IL-6 production is modulated by the glycogen content in muscles [108], which function as an energy sensor [97].

The anti-inflammatory effects of TLR2 and TLR4 during exercise are mediated by the PI3K/AKT/mTOR pathway after an activation of adaptor proteins, leading to the production of IL-10 (Fig. 1) [95], an anti-inflammatory cytokine produced by Th1 cells, monocytes, and macrophages that is present in higher concentrations after physical exercise and acts as a potent inhibitor of pro-inflammatory cytokines [109, 106].

IL-10/IL-10R signaling is mediated by the activation of the JAK/STAT pathway through the phosphorylation of the Tyk2/JAK1 tyrosine, which results in the activation of STAT3 [110]. This mechanism is independent of the toll-like pathway. An analysis of the IL-10/TNF-α ratio is often used as an indicator of inflammatory conditions [32, 111]. This is evidence that IL-10 acts as a natural antagonist of TNF-α and is able to inhibit NF-κβ signaling [110, 112], as shown in Fig. 1. 

## Methods

This review consulted the PubMed database in a search involving seven keywords: “exercise,” “training,” “physical activity,” “TLR,” “TLR2,” “TLR4,” and “toll-like,” To cross-reference the words, 12 groups were created to link terms associated with exercise (“exercise,” “training,” “physical activity”) to toll-like terms (“TLR,” “TLR2,” “TLR4,” and “toll-like”), building groups formed from two individual keywords linked by the Boolean operator “AND.” This produced groups organized as follows: group 1: “exercise” and “TLR”; group 2: “exercise” and “TLR2”; group 3: “exercise” and “TLR4”; group 4: “exercise” and “toll-like”; group 5: “training” and “TLR”; group 6: “training” and “TLR2”; group 7: “training” and “TLR4”; group 8: “training” and “toll-like”; group 9: “physical activity” and “TLR”; group 10: “physical activity” and “TLR2”; group 11: “physical activity” and “TLR4”; and group 12: “physical activity” and “toll-like.”

Only studies carried out directly in animal models (human, rat, and mouse) were included. For scientific substantiation, 119 scientific articles were also consulted in addition to the 39 studies which met the criteria of eligibility for this review.

Criteria which excluded articles from this review, described in Table 1, fell into categories as follows: non-English articles; literature reviews; articles that did not cover Toll-like receptors (TLRs); articles studying TLRs other than TLR2 and TLR4; articles without exercise protocols; experimental articles that did not use humans, mice, or rats; and finally, articles that involved diet, supplementation, or drugs. To do so, codes to link the eligibility criteria of all of the items found in the search were created.

**Table 1:** Eligibility codes

Eligibility codes	Description
I	Included articles
D	Duplicate articles
E1	Non-English articles
E2	Articles that did not provide enough information
E3	Literature review articles
E4	Articles that did not cover Toll-like receptors
E5	Articles studying TLRs other than TLR2 and TLR4
E6	Articles without exercise protocols
E7	Articles that used animal models other than humans, rats, and mice
E8	Articles that involved diet, supplementation, or drugs

**Fig. 2:**
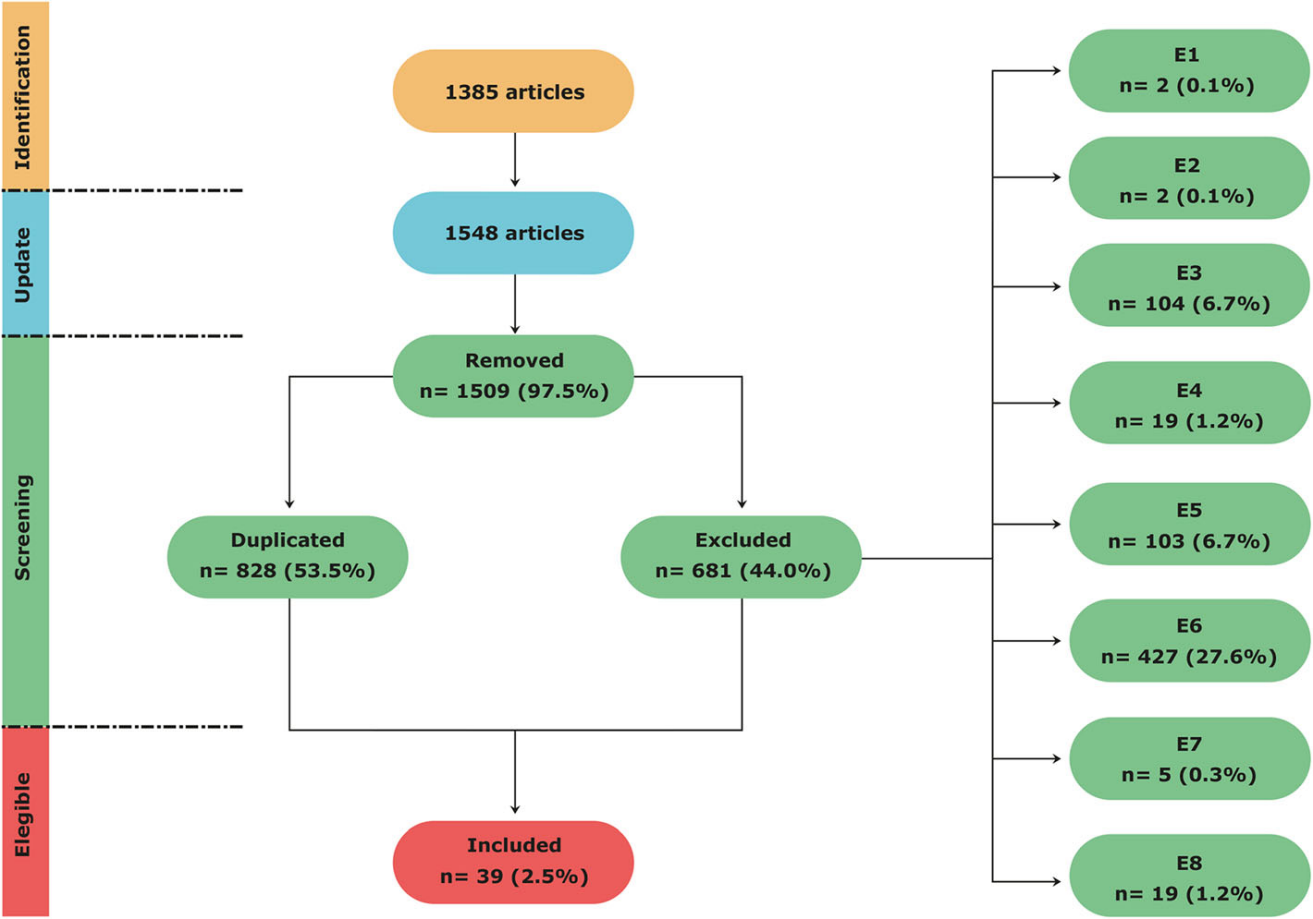
Literature search flowchart

Initially, 1385 articles were found. After an update, the search ended up with 1548 articles from the PubMed database. The updated search was carried out in October 2015. The search group distribution can be seen in Table 2. Figure 2 shows a flowchart of the article selection process, as well as how the articles were linked to the search theme. The total number of articles found and the distribution of the excluded articles are also carefully detailed.

**Table 2:** Distribution of the number of articles per studied groups

Groups	Keywords	Number of articles	Number of articles (after an update)
1	“Exercise” and “TLR”	46	54
2	“Exercise” and “TLR2”	19	23
3	“Exercise” and “TLR4”	64	71
4	“Exercise” and “Toll-like”	111	123
5	“Training” and “TLR”	158	181
6	“Training” and “TLR2”	89	97
7	“Training” and “TLR4”	154	178
8	“Training” and “Toll-like”	372	410
9	“Physical activity” and “TLR”	66	72
10	“Physical activity” and “TLR2”	43	46
11	“Physical activity” and “TLR4”	91	104
12	“Physical activity” and “Toll-like”	172	189
	Total	1.385	1.548

## Results and Discussion

To investigate the roles of TLR2 and TLR4 behavior in the inflammatory and anti-inflammatory effects of exercise, the results were distributed according to the type of exercise (resistance, aerobic, and combined) and frequency of training (acute or chronic), taking the exclusion criteria into account.

Considering the total of 39 studies that met the eligibility requirements for this review, 28 articles were based on the samples from a disease-free setting and 11 samples related to a disease. Three articles studied the effects of exercise and TLR2 and TLR4 on obesity [8, 113, 114], one on pre-diabetes [115], one on low back pain [116], two on cerebral ischemia [13, 14], one on pulmonary inflammation [117], one on Alzheimer’s disease [15], one on chronic fatigue syndrome [36], and one on multiple sclerosis and fibromyalgia [118].

As shown in Table 3, 21 of the 39 eligible articles (54%) showed a reduction in TLR4 and/or TLR2 at the levels of both cell surface protein and mRNA expression, 7 (18%) did not show statistically significant differences, 2 articles (5%) did not test TLR4 and/or TLR2 expression but were included in this review for the evaluation of downstream targets of the receptor pathways, and 9 articles (23%) reported an increase in TLR2 and/or TLR4 (gene expression or protein levels) after AE sessions.

**Table 3:** Results of TLR2 and TLR4 expression of all eligible articles divided by type and frequency of exercise

	Physical exercise	↓TLR2 and 4		↔TLR2 and 4		↑TLR2 and 4		No results		Total
		*n*	%	*n*	%	*n*	%	*n*	%	
Total		21	54	7	18	9	23	2	5	39
Subgroups by exercise types	Chronic resistance	4	67	2	33					6
	Acute resistance	3	75	1	25					4
	Chronic aerobic	7	58	2	17	3	25			12
	Acute aerobic	6	40	1	7	6	40	2	13	15
	Combined	1	50	1	50					2

**Fig. 3:**
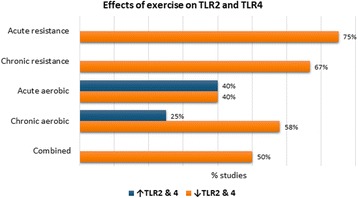
Effects of exercise on TLR2 and TLR4. For chronic resistance exercise, 67% of studies reported a reduction of TLR4 and/or TLR2 expression. For acute resistance exercise, 75% of studies revealed a decrease in the expression of these receptors. For chronic aerobic exercise, 58% of studies reported a reduction in TLR4 and/or TLR2 expression and 25% found an increase in the expression of TLR4 and/or TLR2. For acute aerobic exercise, 40% of studies showed a decrease and 40% reported an increase. Regarding combined exercise, 50% of studies reported a reduction in the expression of the receptors

The results were also analyzed by subgroups and divided according to the type and frequency of training (Table 3 and Fig. 3). For chronic resistance exercise (CRE), four articles (67%) reported a reduction of TLR4 and/or TLR2 expression and two (33%) did not show any significant change. For acute resistance exercise (ARE), three articles (75%) revealed a decrease in the expression of these receptors and one study (25%) failed to find a significant difference. For CAE, seven articles (58%) reported a reduction in TLR4 and/or TLR2 expression, two studies (17%) did not find a significant difference, and three articles (25%) found an increase in the expression of TLR4 and/or TLR2. For AAE, six experiments (40%) showed a decrease, one (7%) did not show any difference, six (40%) reported an increase, and two articles (13%) tested neither TLR2 nor TLR4 expression. Regarding combined exercise (CE), one study (50%) reported a reduction in the expression of the receptors and one study (50%) revealed no significant difference.

### Resistance Exercise and Inflammation

Six articles that studied TLR4 and/or TLR2 behavior with CRE were identified (Table 4). Two studies found a reduction of TLR4 and TLR2 in terms of protein expression [92, 119], two revealed a decrease in mRNA expression [116, 120], and two did not find a statistically significant difference [8, 121]. Three articles [29, 88, 122] showed reductions in the protein and gene expression of TLR4 after an ARE session, and one article [123] did not show a significant difference in TLR2 (protein levels), as shown in Table 5. This systematic review showed that resistance exercise (RE), whether acute or chronic, could act as a regulator of inflammation. In this subset of the literature, we observed no increases in the expression of TLR4 and/or TLR2 or pro-inflammatory cytokines after exercise.

**Table 4:** Modulation of TLR2 and TLR4 after chronic resistance exercise

Authors	Sample	Disease	Frequency, intensity, and duration	Post-exercise results		
				TLR	Cytokine	Other
Zanchi et al. 2010 [120]	Wistar rats	No disease	2 days/week, 80–95% MVSC, 12 weeks	↓TLR4	↓TNF-α ↔IL-6 ↔IL-10 ↑IL-10/TNF-α ratio ↔IL-15	↔Hsp70
Cheng et al. 2015 [116]	Adults	Low back pain	3 days/week, no information, 4 weeks	↓TLR4	↓TNF-α ↓IL-6 ↓IFN-γ ↓IL-1β ↓IL-8	↓NF-kBp65, ↓p53, ↑SIRT1, ↑PGC-1α, ↑PPAR-γ, ↑FoxO1, ↑FoxO3, ↑IKB, ↑SOD
Rodriguez-Miguelez et al. 2014 [119]	Elderly	No disease	2 days/week, 60–80% 1 RM, 8 weeks	↓TLR2, ↓TLR4	↔TNF-α ↑IL-10	↓MyD88, ↓p65, ↓phospho-p38/p38, ↓IKKi/IKKƐ, ↓TRIF, ↓phospho-IRF3/IRF3, ↓phospho-IRF7/IRF7, ↓Hsp60, ↑Hsp70, ↑phospho-ERK1/2, ↓CRP.
Rodriguez-Miguelez et al. 2015 [92]	Elderly	No disease	2 days/week, 20–35 Hz, 8 week	↓TLR2, ↓TLR4	↓TNF-α ↑IL-10	↓MyD88, ↓p65, ↓TRIF, ↓Hsp60, ↑Hsp70, ↓CRP
Prestes et al. 2015 [121]	Elderly	No disease	2 days/week, 6–14 RM, 16 weeks	↔TLR4	↔IL-1β ↔IL-10 ↔IL-1ra ↔ IL-15	↔BDNF, ↔irisin ↑functional capacity, ↑neuromuscular function, ↔body composition
Phillips et al. 2012 [8]	Elderly	Obesity	3 days/week, 8–12 RM, 12 weeks	↔TLR4	↓TNF-α ↓IL-6 ↑IL10	↓CRP, ↓leptin, ↑LPS-IL10, ↑LPS-TNF, ↔body composition

**Table 5:** Modulation of TLR2 and TLR4 after acute resistance exercise

Authors	Sample	Disease	Intensity and duration	Post-exercise results		
				TLR	Cytokine	Other
Millard et al. 2013 [123]	Adults	No disease	120–150 beats/min, 68.8 s (up and down 150 steps)	↔TLR2	↔IFN-γ	↑CD3−/CD56+NK, ↓NK CD56bright Short exercise did not affect NK cytotoxicity.
Fernandez-Gonzalo et al. 2012 [88]	Adults	No disease	40–50 MVIC, 18 acute eccentric bouts	↓TLR4	↓TNF-α	↓CD14, ↓MyD88, ↓TRIF, ↓TRAF6, ↓p65, ↓phospho-IκB, ↓phospho-ERK1/2, ↓CRP, 2 h after the 2nd acute session.
Fernandez-Gonzalo et al. 2014 [122]	Adults	No disease	40–50 MVIC, 18 acute eccentric bouts	↓TLR4	↓TNF-α	↓CD14, ↓MyD88, ↓TRIF, ↓TRAF6, ↓p65, ↓phospho-IκB, ↓phospho-ERK1/2, ↓CRP, 2 h after the 2nd acute session.
McFarlin et al. 2004 [29]	Elderly	No disease	80% 1 RM, 1 bout/3 sets/10 repetitions	↓TLR4	↔TNF-α ↔IL-6 ↔IL-1β	↔CD14

Some studies [30, 124, 125] corroborate the results of this review and suggest that CRE may have anti-inflammatory effects. In contrast, ARE may stimulate changes in metabolic demand and promote inflammatory responses, whose occurrences is fundamentally determined by the exercise protocol [126, 127]. In this analysis, ARE transiently increases circulating levels of CK and pro-inflammatory cytokines, e.g., TNF [126] and IL1β [127]. Some studies that were not eligible for this review [128, 129] have shown that ARE induced microdamage in the skeletal muscle, along with an increase in inflammation markers such as IL-6, IL-8, monocyte chemotactic protein-1 (MCP-1), CK, and CRP when performed at high levels of stress.

The ten eligible studies of CRE and ARE [8, 29, 88, 92, 116, 119–123], tested different frequencies, intensities, and durations of exercise, none of these methods, however, produced changes in levels of TLR2 and/or TLR4. In these studies, intensities ranged from 60 to 80% of 1 RM with a gradual increase [119], or 6–14 RM [121]. In one study [92], the training volume followed a criterion of progression. Another study [120] used 80, 90, and 95% of maximal volitional strength capacity (MVSC), with low training volume as the criterion.

Regarding the inflammation markers that were subjected to the analysis here, neither acute nor chronic RE increased levels of pro-inflammatory cytokines such as TNF-α or IL-6. Eight studies tested TNF-α, and the majority [8, 88, 92, 116, 120, 122] found a significant decline of this cytokine. Two studies [29, 119] found no difference in this marker. Four studies analyzed levels of IL-6 after RE. Two studies [8, 116] found a drop in levels, but no significant difference appeared in the studies by Zanchi et al. [120] and McFarlin et al. [29].

The results showed that the RE protocols for both chronic and acute training adopted by the authors did not generate a pro-inflammatory response. Instead, three studies analyzed by this review [92, 119, 120] established an inverse relationship between the TLR2 and TLR4 receptors and IL-10. In the five studies that investigated IL-10 with RE, four [8, 92, 119, 120] found an increase in this marker and one study found no significant difference [121]. It is known that IL-10 levels are higher after chronic exercise, and this anti-inflammatory cytokine acts as a natural TNF-α antagonist [106, 109].

### Aerobic Exercise and Inflammation

A total of 12 articles verified that TLR4 and TLR2 undergo changes in response to CAE (Table 6). Four studies verified a significant decrease in TLR4 and/or TLR2 [13, 15, 76, 115] in terms of protein levels, two studies [117, 130] showed reductions in mRNA expression, and one indicated decreases at both the gene and protein level [14]. Two studies [74, 114] revealed an increase in TLR4 and/or TLR2 (gene and protein), one study reported increased mRNA expression [131], and two studies [113, 132] did not find any significant difference in TLR4 expression.

**Table 6:** Modulation of TLR2 and TLR4 after chronic aerobic exercise

Authors	Sample	Disease	Frequency, intensity, and duration	Post-exercise results		
				TLR	Cytokine	Other
Ma et al. 2013 [13]	Wistar rats	Cerebral ischemia	5 days/week, 12 m/min, 3 days–2 weeks	↓TLR4, ↓TLR2		↓NFkB e ↓MyD88
Lira et al. 2010 [76]	Wistar rats	No disease	5 days/week, 15–25 m/min, 11 weeks	↓ TLR4 (TR group), ↑TLR4 (R group)	TR group: ↔ TNF-α ↔IL-6 ↔IL-10	TR group (trained) ↓NFkBp65. OT group (overtrained) and R (resting overtrained): ↓performance decline, ↓testosterone, ↑corticosterone, ↑endotox. ↑IL-6, ↑IL-10, ↑NFkBp65
Fashi et al. 2015 [117]	Wistar rats	Pulmonary infection	5 days/week, mean speed of the group workload, 4 weeks	↓TLR4	↓TNF-α	↓NF-kB (exe group+PM10)
Jun et al. 2014 [130]	Sprague-Dawley rats	Ovariectomized rats	5 days/week, 18–26 m/min, 16 weeks	↓TLR4	↓TNF-α ↔IL-6	↓MCP-1 in adipose tissue (moderate trained group).
Holland et al. 2015 [132]	Sprague-Dawley rats	No disease	1/day, 30 m/min, 10 days	↔TLR4	↓TNF-α ↔IL-6 ↔IFNy ↔ IL10	Moderate training: ↔NFkB, ↔CCL2, ↔IL10, ↔NFkBp65
Zwagerman et al. 2010 [14]	Sprague-Dawley rats	Stroke	5 days/week, 30 m/min, 3 weeks	↓TLR4		↓Cerebral infarction volume
Choi et al. 2014 [15]	Sprague-Dawley rats	Alzheimer’s disease	5 days/week, 2–8 m/min, 6 weeks	↓TLR4	↓TNF-α ↓IL-1α	↓NF-kB, in the STZ-exe group. ↑Cognitive function
Zheng et al. 2015 [131]	Adults (members of a university badminton club)	No disease	3 days/week, no information, 26–32 days	↑TLR2, ↔TLR4, with or without microbial antigen stimulation	↑TNF-α ↑IL-6 with or without microbial antigen stimulation	
Robinson et al. 2015 [115]	Adults	Pre-diabetes	1/day, 65–90% peak heart rate, 2 weeks	↓TLR4, ↓TLR2	↔TNF-α ↔IL-6 ↔IL-1β ↔ IL10	↓Fasting glucose in group MICT (moderate-intensity continuous training).
Nickel et al. 2011 [114]	Adults (amateur marathon runners)	Obesity	Training documented with respect to intensity, duration, and kilometers run per week by a written individual protocol, 10 weeks	↑TLR2 in LNE group (lean-non-elite). ↑TLR4 (all groups).	↔TNF-α	↑oxLDL in LE (lean-elite); ↓oxLDL in ONE (obese-non-elite).
Nickel et al. 2012 [113]	Adults (amateur marathon runners)	Obesity	Training documented with respect to intensity, duration, and kilometers run per week by a written individual protocol, 10 weeks	↔TLR2, ↔TLR4	↑TNF-α (24 h post-marathon) ↑IL-6 and ↑IL-10 (immediately after the run)	↑BDCA-1, ↓BDCA2, ↓TLR7, ↑PCR, ↔oxLDL
Ghosh et al. 2015 [74]	Adults and elderly	No disease	3–4 days/week, 65–80% VO2max, 16 weeks	↑TLR4 (aged individuals)		In elderly: ↑NF-kBp65, ↑NF-kBp50, ↑pJNK, ↑endotoxin, ↔pERK, ↔p-p38, ↑insulin resistance.

In 15 studies, a relationship between AAE and TLR2 and/or TLR4 was identified (Table 7). Three studies [133–135] found a significant reduction of TLR4 and/or TLR2 (protein levels), and two revealed a decrease in mRNA expression [136, 137]. Four studies [35, 39, 40, 42] found an increase in the protein levels of these receptors, and two studies [36, 37] increased mRNA expression. One study did not find a significant difference [138], and one study reported a significant decline in TLR4 (mRNA expression) in multiple sclerosis but found no difference in cases of fibromyalgia [118]. Two studies [139, 140] did not analyze TLR2 or TLR4 expression.

**Table 7:** Modulation of TLR2 and TLR4 after acute aerobic exercise

Authors	Sample	Disease	Intensity and duration	Post-exercise results		
				TLR	Cytokine	Other
Rosa et al. 2011 [40]	Wistar rats	No disease	70% VO2max, 50 min	↑TLR4		↑MyD-88, ↑TRAF6, ↑NF-kBp65
Rodriguez-Miguelez et al. 2015 [39]	Wistar rats	No disease	16 m/min, 90 min/18 bouts/5 min/bout	↑TLR4	↑TNF-α ↑IL α-1β	↑HIF-1α, ↑VEGF, ↑eNOS, ↑MPO.
Liao et al. 2010 [136]	Sprague-Dawley rats	No disease	25 m/min, 1–2 h	↓TLR4	↑TNF-α	↑TNF-α, ↑NFkB, ↑p65, ↑ROS, ↑endotoxina
Zbinden-Foncea et al. 2012 [42]	Mice	No disease	70% of FCmax, two bouts of 60 min	↑TLR2, ↑TLR4		↑NEFA, ↑p38MAPK, ↑JNK.
Tanaka et al. 2010 [138]	Mice	No disease	9 m/min to exhaustion, 1 acute bout	↔TLR4	↓TNF-α	
Ortega et al. 2009 [140]	Adults	No disease	70% VO2 max,1 h			Hsp72-induced stimulation of neutrophil chemotaxis disappeared when TLR2 was blocked.
Lancaster et al. 2005 [133]	Adults	No disease	65% VO2max, 1.5 h	↓TLR4, ↓TLR2	↓IL-6	
Booth et al. 2010 [35]	Adults	No disease	60 km distance in the cycle the fastest possible time. Heart rate (bpm) and power output (watts) were monitored	↑TLR2, ↑TLR4		↓HLA.DR
Simpson et al. 2009 [135]	Adults	No disease	75% VO2max, 45 min	↓TLR4, ↓TLR2		↓HLA.DR
Neubauer et al. 2013 [37]	Adults	No disease	Borg 6–20, 10 km	↑TLR4	↑IL-6 ↔IL-1β ↑IL-10 ↑IL-1ra	↑IRAK3, ↑creatin kinase 3 h after, ↑plasma myoglobin 3 h after, ↑neutrophil 3 h after
Oliveira and Gleeson 2010 [134]	Adults	No disease	75% VO2peak, 1.5 h	↓TLR4		TLR4 returned to basal levels within 4 h after exercise, ↔TLR2.
Radom-Aizik et al. 2014 [137]	Adults	No disease	82% VO2peak, 2-min bouts	↓TLR4	↓TNF-α	↓CD36 e ↑EREG genes and ↑CXCR4
Light et al. 2009 [36]	Adults	Chronic fatigue syndrome	70% age-predicted maximal heart rate, 5–9 min	↑TLR4	↑IL6 ↑IL1β ↑IL-10 ↑IL13 ↑IL8 ↑IL12	↑Pain ↑mental fatigue. ↑α2-A, ↑RNAm of β-2 receptor in leucocytes, ↑COMT RNAm
White et al. 2012 [118]	Adults	Multiple sclerosis (ME) and fibromyalgia (SDC)	70% of age-predicted maximal heart rate, 20 min	ME: ↓TLR4 SDC: ↔TLR4	ME: after 8 h ↔IL-6 ↑IL-10 SDC: after 48 h ↔IL-6 ↑IL-10	↑Fatigue ↑pain, ↑adrenergic receptors.
Li and Geib 2013 [139]	Adults and elderly	No disease	1 h Tai Chi		↑IL-13	↓CD14+CD16+

As demonstrated by the results from the analysis of TLR2 and TLR4 behavior, this review showed that in 23% of all of the articles that were analyzed, AE was associated with increases in inflammation. These results differ from previous studies that tested the expression of these receptors in RE. Ten months of CAE was more effective than strength and flexibility exercises in reducing inflammatory markers such as CRP, IL-6, and IL-18 in the elderly [141].

Most studies found that CAE reduced the levels of TLR2 and/or TLR4 [13–15, 76, 115, 117, 130]. However, the major immunological benefits came with exercise performed at a moderate intensity [13–15, 76, 117, 130, 132]. On the other hand, Zheng et al. [131] observed an increase in TLR2 (gene expression) and inflammatory cytokines such as TNF-α and IL-6 in the regular moderate intensity exercise group (badminton), with or without stimulation from microbial antigens. However, cytokine levels were suppressed after non-microbial antigen stimulation. The authors attributed this result to possible improvements in the body’s resistance to invasion by pathogens in response to regular exercise, indicating that an increase of these receptors does not necessarily indicate a negative impact on health, though further research is still needed to address this possibility.

The chronic low-grade inflammatory profile (CLIP) is a common feature of the normal aging process, and it is also involved in the pathogenesis of several age-related diseases [142]. CLIP has already been recognized as a factor that plays a causative role in the development of sarcopenia. TNF-α and IL-6 are the most commonly reported inflammatory parameters in these studies [143]. Additionally, human aging is associated with metabolic endotoxemia and high levels of signaling of the RST4-NFkB-MAPK pathway in the muscle. These factors may play a role in the types of insulin resistance mediated by aging and muscle loss [74]. In this analysis, Ghosh et al. [74] observed an increase in TLR4 (mRNA and protein levels) in older people but not in younger participants. The study examined people engaged in a progressive regime of the intensity and volume of training, ranging from 65 to 80% of VO2max, and an increase in the duration and number of sessions. Their results provide evidence that higher LPS flow in the elderly can play a critical role in age-related sarcopenia and insulin resistance.

Studies that did not fit our criteria [54, 58, 144, 59, 145] suggested that CAE performed under conditions of high stress leads to inflammation in participants of all ages. They observed that long-distance runners might have increased levels of atherosclerosis and coronary heart diseases due to a training regime that went uninterrupted over many years [54]. Additionally, endotoxemia was found in 68% of athletes after a long-distance triathlon, and LPS levels were associated with higher levels of CRP [75]. A recent study showed that 24 h of continuous ultramarathon activity resulted in a higher level of LPS and increased levels of circulating pro-inflammatory cytokines [146]. In fact, prolonged intense physical exercise leads to elevated concentrations of counter-regulatory hormones in plasma such as cortisol and catecholamines related to low immunity [147]. In addition, high levels of muscle oxidative stress lead to an excessive production of ROS and inflammation [60]. In contrast, regular moderate physical exercise can compensate for oxidative stress [148].

Short acute sessions of physical exercise may disturb homeostasis and increase inflammation [41], as verified by some of the articles reviewed here [35, 37, 39, 40, 42]. With the exception of the study by Light et al. [36], which tested an AAE protocol at moderate intensity and in samples obtained from individuals with disease, studies based on different strenuous exercise protocols consistently led to increases in TLR4, TLR2, and pro-inflammatory cytokines [35, 37, 39, 40, 42]. Rodrigues-Migueles et al. [39] found an increase in TLR4 (protein) and pro-inflammatory cytokines in AAE sessions. However, all of these effects were extinguished by CAE through a weekly exercise protocol of increasing intensity and duration.

In studies which reported increases in TLR2, TLR4, and pro-inflammatory cytokines after acute sessions, IL-10 was tested in only three experiments, all of which revealed a significant increase in the expression of this cytokine [36, 37, 118]. This was probably caused by a transient increase in IL-6 which then led to a subsequent increase in levels of IL-10 [104, 106]. However, other studies [133–135] indicated that AAE had beneficial effects, as observed through a decline in terms of protein levels of TLR2 and/or TLR4 and at the mRNA expression [118, 137]. Radom-Aizik et al. [137] verified that AAE not only prevents the normal effects of aging in terms of atherosclerosis but also reduces its symptoms in a manner that promotes cardiovascular health despite the global stress response that is generally evoked by this activity.

One exception is a study by Liao et al. [136], which showed a reduction in TLR4 (gene expression), but also showed an increase in inflammatory responses as exhibited by high levels of TNF-α, NF-kB, and LPS. The reason for the down-regulation of TLR4 is not clear, but the authors believe that this may be related to high levels of ROS. Here, from our review of the literature, we suggest that increases in circulating LPS and an excessive generation of ROS are the main actors in the acute inflammatory process generated by excessive AE. However, more studies are needed to complete the mechanistic picture that links these effects and other aspects of inflammatory responses in AE.

### Combined Exercise and Inflammation

Only two studies [93, 149] relating TLR2 and/or TLR4 to CE (combining aerobic and resistance exercises in single sessions) were found. One study [93] demonstrated a significant decline in TLR4, and the other [149] did not find a difference in TLR4 (Table 8).

**Table 8:** Modulation of TLR2 and TLR4 after combined exercise (aerobic and resistance)

Authors	Sample	Disease	Frequency, intensity, and duration	Post-exercise results		
				TLR	Cytokine	Other
Stewart et al. 2005 [93]	Adults and elderly	No disease	3 days/week, 70–80% 1 RM and 50–70% of heart rate reserve, 12 weeks	↓TLR4, ↔TLR2	↔TNF-α ↔IL-1β ↓IL-6	
Timmerman et al. 2008 [149]	Elderly	No disease	3 days/week, 70–80% 1 RM and 70–80% of heart rate reserve, 12 weeks	↔TLR4	↓TNF-α	

The Timmerman et al. [149] study analyzed the response of 12 weeks of exercise training on the part of aged, physically inactive subjects who performed AE for 20 min and RE for 30 min. No significant differences in TLR4 (protein expression) were found in the trained group compared to the controls, but a decline in TNF-α was observed. Stewart et al. [93] compared CE effects in adult and aged participants and showed a significant decline in TLR4 as well as IL-6 in the physically inactive groups compared to controls; however, levels of TLR2 were not significantly changed.

Another experiment [150] verified a decline in CRP in both trained and active control groups and concluded that AE and RE may be applied in the same session as a potential therapeutic intervention for adults and aged individuals to avoid some chronic diseases. Therefore, this review suggests that AE and RE in combination protect against the negative effects of AE.

### Exercise, Disease, and Inflammation

The majority of the studies eligible for this review show that both AE [13–15, 113–115, 117] and RE [8, 116] can act as excellent auxiliary treatments for chronic disease. However, we found no article that tested ARE in samples from patients with diseases.

One of the important features of obesity-induced inflammation is a phenotypic change in the populations of macrophages and T cells present in the adipose tissue. This is reflected in levels of the production of anti- and pro-inflammatory cytokines [151]. It has been suggested that free saturated fatty acids can induce inflammation through the activation of macrophages, TLR2, and TLR4 in the adipose tissue, culminating in the activation of NF-kB and an increased expression of pro-inflammatory cytokines such as TNF-α or IL-6 [7, 9, 151].

The study by Phillips et al. [8] in post-menopausal obese women showed that CRE did not decrease TLR4 in terms of mRNA expression but reduced inflammatory markers such as TNF-α and IL-6. In another study related to obesity, 10 days of either moderate (MICT) or high intensity (HIIT) CAE in inactive overweight women promoted improvements in glucose control and cardiorespiratory capacity and a decrease in TLR2 and TLR4 (protein content) [115].

Most studies in this review that tested the levels of TLR2 and/or TLR4 receptors in a disease context used moderate load protocols, with the exception of the study by Nickel et al. [114], which studied marathon runners and found an increase in the mRNA expression and protein levels of these receptors. In this study, TLR2 was significantly increased in lean-non-elite athletes when compared to the obese-non-elite and lean-elite groups, and TLR4 increased in all groups in response to exercise. However, levels of the systemic cytokines TNF-α and IL-6 remained stable. Interestingly, oxidized low-density lipoprotein (oxLDL) levels in obese athletes were reduced and associated with higher adiponectin levels, in contrast to increased levels of oxLDL found in the group of lean-elite athletes [114]. This can be understood from the fact that TLR4 plays a crucial role in cellular responses to oxLDL exposure and the activation of NF-κB [152, 153]. Wang et al. [152] showed that the activation of the TLR4/NF-κB signaling pathway was a potential mechanism for oxLDL-induced apoptosis in cardiomyocytes.

Higher levels of this low-density lipoprotein (LDL) are usually associated with an increased risk for atherosclerosis [114], and marathon runners may, in fact, have increased levels of atherosclerosis [54]. LDL, when modified by enzymes such as phospholipases, gives rise to oxidized low-density lipoprotein (oxLDL), which contributes to the formation and progression of atherosclerotic plaques [152, 154]. oxLDL is known to be immunogenic and activates endothelial cells, monocytes, macrophages, and T cells [155]. Furthermore, oxLDL is toxic at higher concentrations and thus could be a cause of cell death in lesions [156]. The plasma level of oxLDL was shown to be a predictor of mortality in patients with chronic congestive heart failure [157] and induced severe cell damage in ventricular myocytes [158].

This review also found articles that generally analyzed TLR2 and/or TLR4 expression in relation to other diseases. The study by Zwagerman et al. [14], for example, found that in addition to reduced levels of TLR4 (gene and protein), CAE reduced the frequency of cerebral infarction. Another study [36] analyzed chronic fatigue syndrome in acute AE sessions at moderate intensity for 25 min. In addition to an increase in the mRNA expression of TLR4 and pro-inflammatory cytokines, symptoms such as pain and physical and mental fatigue became worse after exercise, suggesting a dysregulation of the immune and sympathetic nervous systems.

## Conclusions

This is the first systematic review of the literature that addresses the roles of TLR2 and TLR4 receptors in various types of exercise. Our main finding is evidence for an accentuation in the inflammatory processes orchestrated by these receptors in both AAE and CAE. The results also suggest that the expression of the receptors is correlated with that of anti- and pro-inflammatory cytokines. Taken together, these data open new perspectives for studies aimed at a better understanding of the response of inflammatory processes to physical exercise.

An analysis of the pathways involving TLR2 and TLR4 reveal something about the way specific types of physical exercise are related to differences in the types of inflammatory responses they stimulate. The results indicate that AE is potentially inflammatory; a smaller number of studies revealed that acute exercise has anti-inflammatory effects, compared to studies of chronic exercise.

Our analysis showed that in RE, TLR2 and TLR4 expression and signaling adopt an anti-inflammatory pattern. Studies that met our criteria for inclusion indicated that acute or chronic sessions reduced TLRs as well as inflammatory cytokines, particularly TNF-α, and promoted increases in IL-10, which can be considered a beneficial adaptation for both healthy people and those affected by certain diseases.

The same results were obtained when differences in the populations and intensities of exercise were taken into account. This indicates that RE can be broadly used to prevent or minimize the potentially deleterious effects of TLR expression and that the intensity can be manipulated to achieve other goals, such as increasing body strength, without a loss of benefits vis-à-vis the overall inflammatory profile.

For AE, the intensity of exercise is a crucial factor—better responses were achieved under moderate intensities. But overall, whether the effects of AE will be positive or negative depends on a person’s other physiological characteristics, so they must be taken into account.

Generally, CE seems to be a good choice in most situations due to its positive effects on TLR expression and signaling. In other words, the possible negative “side effects” of AE can be overcome through the positive impact of RE. This combination of training strategies appears to improve a person’s general inflammatory profile while maintaining the cardiovascular and metabolic benefits of AE. In most cases, this leads to better adaptations. But because the number of studies addressing the effects of TLR2 and TLR4 in CE is very small, further research is needed for both amateurs and elite athletes.

## Abbreviations

AAE: Acute aerobic exercise
AE: Aerobic exercises
ARE: Acute resistance exercise
Arg1: Arginase-1
CAE: Chronic aerobic exercise
CE: Combined exercise
CK: Creatine kinase
CRE: Chronic resistance exercise
CRP: C-reactive protein
DAMPs: Damage-associated molecular patterns
IGF-1: Insulin-like growth factor 1
LPS: Lipopolysaccharides
MAPK: Mitogen-activated protein kinase
PAMPS: Pathogen-associated molecular patterns
RE: Resistance exercise
ROS: Reactive oxygen species
TLR: Toll-like receptor
TLR2: Toll-like receptor 2
TLR4: Toll-like receptor 4
TNF-α: Tumor necrosis factor alpha
VEGF: Vascular endothelial growth factor
