# Correction to: Aerobic but not Resistance Exercise Can Induce Inflammatory Pathways via Toll-Like 2 and 4: a Systematic Review

**DOI:** 10.1186/s40798-018-0120-9

**Published:** 2018-01-31

**Authors:** P. A. M. Cavalcante, M. F. Gregnani, J. S. Henrique, F. H. Ornellas, R. C. Araújo

**Affiliations:** 1 Medicine (Nephrology) Program, Federal University of São Paulo (UNIFESP), São Paulo, SP Brazil; 2 Laboratory of Exercise Genetics and Metabolism, Federal University of São Paulo (UNIFESP), São Paulo, SP Brazil; 3 Department of Biophysics, Federal University of São Paulo (UNIFESP), São Paulo, SP Brazil; 4 Rua Pedro de Toledo, 669/9and., 04039-032, São Paulo, SP Brazil; 5 Molecular Biology Program, Federal University of São Paulo (UNIFESP), São Paulo, SP Brazil; 6 Laboratory of Exercise Genetics and Metabolism, Federal University of São Paulo (UNIFESP), São Paulo, SP Brazil; 7 Department of Biophysics, Federal University of São Paulo (UNIFESP), São Paulo, SP Brazil; 8 Neurology/Neuroscience Program, Federal University of São Paulo (UNIFESP), São Paulo, SP Brazil; 9 Exercise Neurophysiology Laboratory, Federal University of São Paulo (UNIFESP), São Paulo, SP Brazil; 10 Medicine (Nephrology) Program, Federal University of São Paulo (UNIFESP), São Paulo, SP Brazil

## Erratum

The original article [1] mistakenly omits a grant acknowledgement; thus, the authors would like to acknowledge that the original article was supported by FAPESP 2015/20082-7.
